# Recent advances in the development of gene delivery systems

**DOI:** 10.1186/s40824-019-0156-z

**Published:** 2019-03-12

**Authors:** YK Sung, SW Kim

**Affiliations:** 1 Department of Pharmaceutics and Pharmaceutical Chemistry, University of Utah, Salt Lake City, UT 84112 USA; 2 Center for Controlled Chemical Delivery, University of Utah, Salt Lake City, UT 84112 USA; 3 Department of Chemistry, Dongguk University, Chung-gu, Seoul 04620 Korea; 4 Center for Controlled Chemical Delivery (CCCD), Department of Pharmaceutics and Pharmaceutical Chemistry, University of Utah, BPRB, Room 205, Salt Lake City, UT 84112 USA

**Keywords:** Gene delivery system, Viral vectors, Non-viral vectors, RNA, DNA

## Abstract

**Background:**

Gene delivery systems are essentially necessary for the gene therapy of human genetic diseases. Gene therapy is the unique way that is able to use the adjustable gene to cure any disease. The gene therapy is one of promising therapies for a number of diseases such as inherited disorders, viral infection and cancers. The useful results of gene delivery systems depend open the adjustable targeting gene delivery systems. Some of successful gene delivery systems have recently reported for the practical application of gene therapy.

**Main body:**

The recent developments of viral gene delivery systems and non-viral gene delivery systems for gene therapy have briefly reviewed. The viral gene delivery systems have discussed for the viral vectors based on DNA, RNA and oncolytic viral vectors. The non-viral gene delivery systems have also treated for the physicochemical approaches such as physical methods and chemical methods. Several kinds of successful gene delivery systems have briefly discussed on the bases of the gene delivery systems such as cationic polymers, poly(L-lysine), polysaccharides, and poly(ethylenimine)s.

**Conclusion:**

The goal of the research for gene delivery system is to develop the clinically relevant vectors such as viral and non-viral vectors that use to combat elusive diseases such as AIDS, cancer, Alzheimer, etc. Next step research will focus on advancing DNA and RNA molecular technologies to become the standard treatment options in the clinical area of biomedical application.

## Background

Gene therapy is the unique technique that uses gene to prevent or recover any diseases. The technique of gene therapy may allow doctors to treat a disorder by inserting a gene into patient’s cell instead of using drugs or surgery. Some researchers and doctors are examining several approaches to gene therapy, including: i) replacing a mutated gene that causes disease with a healthy gene; ii) ‘knocking out’ or inactivating, a mutated gene that is functioning improperly; and iii) introducing new genes into the cells to protect from any diseases.

Although gene therapy is a promising treatment option for a number of diseases such as cancer, inherited disorders and certain viral infections, the technique remains risky and is still under examination to make safe until it will be effective. The gene therapy has currently examined only for diseases that have no other cure techniques. Genetic molecules reach to the nuclei of host cells to induce gene expression [1–3]. Gene therapy has derived to provide a patient’s somatic cells with genetic information for producing specific therapeutic proteins to modulate genetic diseases. In order to get a successful design of gene delivery system, it must be required the complete understanding of interaction between targeting cell and gene delivery system. The gene delivery systems consist of the three components such as a plasmid-based gene expression system that controls the function of a gene within the targeting cell, a gene that encodes a specific therapeutic protein, and a gene delivery system that controls the delivery of the gene expression plasmid to specific location within the body [4, 5]. The successful gene delivery system requires the foreign genetic molecule to remain stable within the host cells [6–8]. As a tool of transgene expression, viral-based vectors had firstly emerged in the 1980’s [9]. The virus as a vaccine vector was used the vaccinia virus in 1984 as a way to protect chimpanzees against Hepatitis B [9]. On the other hand, non-viral gene delivery system had firstly reported on showing cellular phenotype change via DNA exposures in the body [10, 11].

In this article, the recent advances in the development of gene delivery systems have reviewed, including the progress of viral gene delivery systems and non-viral gene delivery systems. The viral gene delivery systems have discussed for the viral vectors based on DNA, RNA, and oncolytic viral vectors, respectively. The non-viral gene delivery systems have also treated for the physicochemical approaches such as physical method and chemical method. Several kinds of gene delivery systems have discussed on the gene delivery systems of cationic biomedical polymers such as polysaccharides, polyethylenimine (PEI), and poly(L-lysine)(PLL) derivatives.

### Development of gene delivery systems

#### Viral vector gene delivery systems

Viruses mediated gene delivery systems utilize the ability of virus to inject their DNA inside host cells. Those take the advantage of the virus ability to replicate their own genetic materials. Virus is an effective form of gene delivery because of the virus structure preventing degradation via the lyposome of DNA [12–14]. In gene delivery systems, the genes open to intend for delivery system package into the replication. The deficient viral particles form the viral vectors [15, 16]. Viruses used for gene delivery systems include retrovirus, adenovirus, adeno-associated viruses, and herpes simplex viruses. There are two categories of gene delivery systems such as the germline gene delivery systems and the somatic gene delivery systems. Although the germline gene delivery systems may have great potential, the germline gene therapy cannot use ethically [17, 18]. In practice, gene delivery system for human has been only limited to somatic cell alteration. In somatic gene delivery system, there are three types such as in vivo delivery, ex vivo delivery and in situ delivery. In vivo delivery systems, the genetic materials transfer directly into the targeting tissue. In ex vivo delivery system, the genetic materials implant into the targeting tissue bone marrow, cultivated, and manipulated in vitro*.* The genetic materials transfer into the targeting tissues. There are no immunologic responses in the way but only the techniques use in the various cases. In addition, only a small percentage of implanted cells remain viable at present [19, 20]. In situ delivery system, the administration of the genetic material goes directly into the target tissue. As most of the current delivery systems need no effective targeting, the way is proper [21–23]. The technique is the least advanced strategy at present, but it might potentially be useful method.

##### Viral vectors based on DNA for gene delivery systems

Viral vectors based on DNA for gene delivery are usually longer lasting and integrating into the genomes. DNA-based viral vectors are lentivirus, poxvirus, adenovirus, adeno-associated virus, retrovirus, human foamy virus (HFV) and herpes virus [24]. DNA-based viral vectors for gene delivery systems utilize the viral vectors to deliver genetic materials to the host cells. The viral vectors are efficient for delivering the genetic materials to the host cells [25]. The DNA-based viral vectors for the gene delivery systems include plasmids containing transgenes for gene therapy [26]. Although the DNA-based viral vectors are in early stages of clinical trials, the class of materials has been emerged to yield extremely promising candidate of gene delivery systems for gene therapy in the wide range of diseases including cancer, AIDS, neurological disorders such as Parkinson’s and Alzheimer’s diseases, and cardiovascular disorders [27–29].

##### Viral vectors based on RNA for gene delivery systems

Viral vectors based on RNA for gene delivery have developed for the ability to transcribe directly for infectious RNA transcripts. RNA-based gene delivery is not permanent and usually transient. In the gene delivery systems based on RNA, human foamy virus, oncoretro-viral vectors, and lenti-viral vectors are included in the gene therapy. The sophisticated system provides the RNA-dependent polymerase complexes coupled with negative-strand RNA templates [30]. RNA-based gene delivery systems have carried out for HIV with lenti-viral vectors-modified CD 34(+) cells in patients undergoing transplantation for AIDS-related lymphoma [31].

##### Oncolytic viral vectors for gene delivery systems

Oncolytic viruses (OVs) are an emerging treatment option for cancer type diseases. They have recently focused on cancer research aiming to develop their therapeutic treatment potential [32]. The positive and negative effects of the multitude for their modification have discussed to increase their infectivity, anti-tumor immunity and treatment safety for the interaction of OVs and tumor cells. Oncolytic adenovirus co-expressing IL-12 and IL-18 improves tumor-specific immunity via differentiation of T cells expressing IL-12R**β**_**2**_ or IL-18R**α** [33]. The ultimate aim is to design a virus that is able to replicate effectively within the host, special target and lyse tumor cells. Adenovirus-mediated decorin expression induces cancer cell death through activation of p53 and mitochondrial apoptosis [34]. Oncolytic adenovirus (Ad) vectors present a promising modality to treat cancer by using the gene therapy [35]. Oncolytic adenovirus expressing IL-23 and p35 elicits IFN-**ɤ**-and TNF-α-Co-producing T cell-mediated antitumor immunity. It has reported that cytokine immune-gene therapy is one of promising strategies for cancer treatments [36–39].

#### Non-viral vector gene delivery systems

The physicochemical approaches have recently proposed for non-viral based delivery systems [40]. Non-viral vectors of delivery systems for gene therapy are less likely to induce an immune response in the system of biocompatible materials such as lipid, naked DNA, chromosomes, plasmid, cationic polymers, and conjugate complexes [41].

Non-viral gene delivery mediated artificially by physical method uses to introduce genetic materials through the cell membranes. The physical method of gene delivery includes needle injection, ballistic DNA injection, sono-poration, photo-poration, magneto-fection, and hydro-poration. Needle injection is the direct inject of genetic materials using a needle. On the other hand, ballistic DNA injection is the gold coated DNA articles that force into cells. Electroporation is the electric pulse that creates pores in a cell membrane to allow entry of genetic materials. Sono-poration is the sound wave that creates pores in a cell membrane to allow entry of genetic materials. Photo-poration is the Laser pulse that creates pores in a cell membrane to allow entry of genetic materials. Magneto-fection is the magnetic particles that are complexed with DNA and an external magnetic field, and then they concentrate nucleic acid particles into target cells. Finally, the hydro-poration is the hydrodynamic capillary effect that manipulates cell permeability. The non-viral chemical methods for gene delivery systems use synthetic or natural compounds to form some particles that facilitate the transfer of genes into the cells. The synthetic vector has an ability to interact with RNA or DNA electrostatically and bind to compact the genetic information in the accommodation of larger genetic transfers [42]. Then, non-viral chemical vectors are able to enter cells by endocytosis. In general, there are two non-viral vectors such as liposomes and polymers. Liposome based non-viral vectors use liposomes to facilitate gene delivery by the formation of lipoplexes. The lipoplexes form spontaneously when the negatively charged DNA contacts with positively charged liposomes. The polymer-based non-viral vectors use the polymers to interact with DNA to form polyplexes.

### Practical application of gene delivery systems

A successful gene therapy needs a necessary achievement to develop the proper gene delivery system. The gene delivery system in therapeutic setting utilizes non-immunogenic vectors capable of cell specificity that is able to deliver an adequate amount of transgene expression to cause the desired effect [43]. DNA or RNA microarrays used in a variety of gene sequencing can identify the thousands of genes with analytical software looking at the gene expression patterns [44]. The gene delivery system has utilized to generate hybrid biosynthetic vector to deliver a possible vaccine as an application method. The vector overcomes the traditional barriers to gene deliveries by combining *E. coli* with synthetic polymers to create some vectors [45]. The gene therapy system gives a great opportunity for treating some diseases from genetic disorder, cancer, and other infections. The non-viral vector has a merit in its low immunogenicity and rather low cost reproducibility [46]. The non-viral vector has no limitation in DNA size for packing and modification with ligands to specific cell targeting [47].

The gene delivery systems based on synthetic polymeric materials have tested and evaluated for gene transfer to animals [48]. One of the candidates is the plasmid DNA that is able to carry the gene into the nucleus of the desired cells safely. The gene delivery systems based on non-viral vectors have also risen as a new promising therapeutic modality [49].

#### Gene delivery systems based on cationic polymers

In the research of gene delivery systems, the potential benefits of polymeric carriers for genes have evaluated by various investigators for cationic biopolymers such as liposomes and chitosan derivatives [50, 51]. Regarding to cationic polymers for the gene delivery, the polymeric systems can mask the negative DNA charges and condense the large genes into the small molecular structures. The cationic polymer-based nucleic acid is a complex called as polyplex. The cationic non-viral lipid-based gene carriers, ‘lipoplexes’ have been currently evaluated clinically [52–56]. The gene complex is one of key targeting delivery for gene therapy. Most researches focus on the effect of targeting ligands that are coordinated covalently to the DNA complex. The cationic polymers can conjugate to the targeting ligands. One of candidate is poly(L-lactide), which has widely utilized as a polymer for attaching the targeting ligands [57–60]. The plasmid DNA condensation of cationic polymer undergoes a compaction under the wide variety of conditions. The multivalent cationic polymers have usually used as the condensing agents [61, 62]. The tertiary structures similar to the non-condensing plasmid DNA have been investigated on the complexed with low hydrophobized stearyl-poly(L-lactide) [63]. The DNA condensation has still allowed for the systems of attaching hydrophilic segments such as polyethylene glycol, dextran, hyaluronic acid or hydrophobic stearyl chains [64–67].

#### Gene delivery systems based on polysaccharides

A range of biodegradable cationic polymers based on grafted oligo-amine residues for natural polysaccharides was reported [68]. The grafting idea, in which side chain oligomers have attached to either linear or branched hydrophilic polysaccharides, allows three-dimensional interaction with anionic surfaces of double strand DNA chains. The gene delivery system based on polysaccharide indicates that the structure of the cationic polysaccharide has a significant role in the transfection activity for gene delivery. The colloidal polysaccharide particles had investigated for the mucosal delivery of complexes such as peptides, proteins, oligonucleotides, and plasmids [69]. Natural polysaccharide-based nano-particles had also studied as gene and drug delivery systems [70]. The mechanism was proposed to prepare polysaccharide-based nanoparticles, including covalent and ionic crosslinking, electrolyte complexing, and the self-assembly of hydrophobic polysaccharides. The natural polysaccharides such as chitosan and pectin had studied as gene delivery or drug delivery carriers [71]. Chitosan families have strongly recommended poly-cationic derivatives of poly-N-acetyl-D-glucosamine, which is the positive charge molecule with important muco-adhesion. Pectin is also made of several structural segments, which are homo-galacturonan (HG) rhamnogalaturonan (RG-I) as smooth and hairy regions.

#### Gene delivery systems based on poly(ethylenimine)s

Cationic polymers used for gene complexation are mainly polyamines that become cationic at physiologic conditions. Poly(ethylenimine)(PEI) consists of many nitrogen atoms including primary, secondary, and tertiary amine groups. Those amine groups are able to enhance their buffering capacities. Those cause the subsequent endosomal or lysosomal rupture, and escape into the cytoplasm via a proton sponge effect [72, 73]. PEI is able to provide some buffering capacity enhanced to the other functional molecules. PEI conjugated poly(crystamine bis(acrylamide)-diaminohexane)[poly(CBA-DAH)] had been synthesized to decrease weight ratio and increase transfection efficiency. Poly(CBA-DAH) is composed of multiple disulfides, which can be cleaved in the cytoplasm by intracellular reducing agent such as glutathione(GSH) [74–77]. GSH is composed of triple-peptide, which has synthesized in the cytosol from precursor amino acids [78]. The design of PEI-conjugated bio-reducible polymer for efficient gene delivery had investigated to confirm the successful vectors and polyplexes formation with pDNA. The other studies had also shown the enhanced buffering capacity such as imidazole, histidine, and PEI [79–83]. Biodegradable Poly(ethylenimine)s for plasmid DNA Delivery had been investigated [84]. Branched poly(Ethylenimine)-cholesterol water-soluble lipo-polymers for gene delivery was also developed [85]. Modified Linear Polyethylenimine-Cholesterol Conjugates for DNA Complexation had made for gene delivery [86]. Tumor efficacy and biodistribution of linear polyethylenimine-cholesterol/DNA complexes had tested for gene delivery system [87]. Polyethylenimine with acid-labile linkages as a biodegradable gene carrier had synthesized and tested for gene delivery system [88]. Reducible poly(amidoethylenimine)s designed for gene delivery systems had been made and tested for gene delivery [89–92].

#### Gene delivery systems based on poly(L-lysine)

Poly(L-lysine)(PLL) is one of the cationic polymers for gene transfers. PLL is a homo-polypeptide belonging to the group of cationic polymers at pH 7. PLL contains a positively charged hydrophilic amino group. Replica particles (RP) based on PLL cross-linked via a homo-bi-functional linker to support co-adsorption of a plasmid DNA has designed for gene delivery system [93]. The stabilization of poly-L-lysine/DNA polyplexes for gene delivery in vivo to the liver has been developed [94]. The synthesis and characterization of poly-L-lysine-based gene delivery systems have been reported [95]. N-terminal modified poly(L-lysine)-antibody conjugate had been used as a carrier for targeted gene delivery in mouse lung endothelial cells [96]. Biodegradable poly(ethylene glycol)-*co*-poly(L-lysine)-*g*-histidine multi-block copolymers had been used for non-viral gene delivery systems [97]. Poly(L-lysine) and copolymers for gene delivery had been treated and summarized in polymeric gene delivery [98]. Poly-L-lysine-graft-PEG-comb-type polycation copolymers had also developed for gene delivery systems [99]. Nano-particle DNA carrier with poly(L-lysine) grafted polysaccharide copolymers and terplex DNA delivery system as a gene carrier were firstly developed and characterized [100, 101]. A new non-viral DNA delivery vectors of the terplex systems and polyethylene glycol-grafted poly(L-lysine) as polymeric gene carriers were also developed for gene delivery systems [102–105]. The clinical evaluations have currently made in the field of biomedical applications [106–117].

## Conclusion

An important goal for the research of gene delivery system is to develop clinically relevant vectors that use to combat elusive diseases such as AIDS, cancer, Alzheimer, etc. DNA-based viral vectors for gene delivery systems utilize the viral vectors to deliver genetic materials to the host cells. The DNA viral vectors are efficient for delivering the genetic materials to the host cells. On the other hand, one of promising RNA vectors has established employing naked syn-mRNA, viral or non-viral RNA embedded within viral particles, as well as self-amplifying RNA replicons. RNA-based systems appear superior to common DNA-based modalities. RNA technologies have especially matured in terms of immunogenicity, RNA stability, their formulation and production. Improvement of these parameters has created promising gene transfer systems suitable for in vivo treatment. Next step research will probably focus on advancing DNA and RNA molecular technologies to become standard treatment options in the biomedical applications.
